# Cytonuclear Coordination Is Not Immediate upon Allopolyploid Formation in *Tragopogon miscellus* (Asteraceae) Allopolyploids

**DOI:** 10.1371/journal.pone.0144339

**Published:** 2015-12-08

**Authors:** Tina Sehrish, V. Vaughan Symonds, Douglas E. Soltis, Pamela S. Soltis, Jennifer A. Tate

**Affiliations:** 1 Institute of Fundamental Sciences, Massey University, Palmerston North, New Zealand; 2 Department of Biology, University of Florida, Gainesville, Florida, United States of America; 3 Florida Museum of Natural History, University of Florida, Gainesville, Florida, United States of America; 4 Genetics Institute, University of Florida, Gainesville, Florida, United States of America; Ben-Gurion University, ISRAEL

## Abstract

Allopolyploids, formed by hybridization and chromosome doubling, face the immediate challenge of having duplicated nuclear genomes that interact with the haploid and maternally inherited cytoplasmic (plastid and mitochondrial) genomes. Most of our knowledge of the genomic consequences of allopolyploidy has focused on the fate of the duplicated nuclear genes without regard to their potential interactions with cytoplasmic genomes. As a step toward understanding the fates of nuclear-encoded subunits that are plastid-targeted, here we examine the retention and expression of the gene encoding the small subunit of Ribulose-1, 5-bisphosphate carboxylase/oxygenase (Rubisco; *rbcS*) in multiple populations of allotetraploid *Tragopogon miscellus* (Asteraceae). These polyploids formed recently (~80 years ago) and repeatedly from *T*. *dubius* and *T*. *pratensis* in the northwestern United States. Examination of 79 *T*. *miscellus* individuals from 10 natural populations, as well as 25 synthetic allotetraploids, including reciprocally formed plants, revealed a low percentage of naturally occurring individuals that show a bias in either gene (homeolog) loss (12%) or expression (16%), usually toward maintaining the maternal nuclear copy of *rbcS*. For individuals showing loss, seven retained the maternally derived *rbcS* homeolog only, while three had the paternally derived copy. All of the synthetic polyploid individuals examined (S_0_ and S_1_ generations) retained and expressed both parental homeologs. These results demonstrate that cytonuclear coordination does not happen immediately upon polyploid formation in *Tragopogon miscellus*.

## Introduction

Allopolyploidy is a major mode of plant speciation and results from the union of two or more diverse, but generally closely related, genomes by hybridization and genome duplication [1, 2]. Genomic data indicate that all angiosperms may be regarded as polyploid, if paleopolyploid events are taken into account [3–5]. Allopolyploid genomes experience both immediate and long-term evolutionary changes, which may involve a variety of genetic and epigenetic interactions leading to genome alteration, regulatory incompatibilities, chromosomal abnormalities, and reproductive challenges [6–14]. Polyploidy has been considered a driver of modifications in gene function, potentially resulting in four fates for the duplicated genes (homeologs): (I) both copies are preserved and retain their original functions, (II) one copy maintains the original function whereas the other copy is silenced, or (III) the two copies diverge such that each copy contributes only a part of the original gene function (subfunctionalization) or (IV) one copy attains a novel function (neofunctionalization) [15–21].

In newly formed allopolyploids, coordination between the haploid maternally inherited cytoplasmic (plastid and mitochondrial) and the duplicated biparentally inherited nuclear genomes is required to facilitate genomic stability [8]. Indeed, ‘cytonuclear interactions’ are considered responsible for post-zygotic hybrid incompatibilities and speciation [22–24] and have also caused striking differences in floral traits in reciprocal diploid hybrids [25, 26]. Cytonuclear coordination may also be a contributor to the directional genomic changes and preferential expression of some genes in reciprocally formed polyploids [27–29].

Recent studies of Rubisco (Ribulose-1,5-bisphosphate carboxylase/oxygenase), which comprises a nuclear-encoded subunit (rbcS) and a chloroplast-encoded subunit (rbcL), in allopolyploids have revealed a dynamic nature to the evolution of the nuclear component [30, 31]. In several allopolyploid systems, the duplicated nuclear gene copies (homeologs) of *rbcS* undergo gene conversion in favor of maintaining the maternally derived copy even when the parental chloroplast sequences of *rbcL* are not divergent [30]. Additionally, a common feature of this system is that allopolyploids show preferential expression of the maternal *rbcS* homeolog when both copies are maintained in the genome [30, 31]. How early following polyploid formation this cytonuclear coordination might be established is not known as the polyploids studied to date are several hundred thousand to several million years old [30, 31].

An excellent model system for studying the early stages of allopolyploid cytonuclear coordination is offered by *Tragopogon* (Asteraceae). Following the introduction of three diploid species from Europe (*Tragopogon dubius*, *T*. *pratensis*, and *T*. *porrifolius*) to the Palouse region of eastern Washington State/western Idaho, USA, in the early 1900s, two allopolyploid species were formed. *Tragopogon mirus* (*T*. *dubius* × *T*. *porrifolius*) and *T*. *miscellus* (*T*. *dubius* × *T*. *pratensis*) both formed repeatedly in the past 80 years in western North America with *T*. *miscellus* also forming reciprocally, yielding short-liguled (*T*. *dubius* ♂ × *T*. *pratensis* ♀) and long-liguled (*T*. *dubius* ♀ × *T*. *pratensis* ♂) forms [32–34]. Recurrent formation of both allopolyploids and restricted gene flow among origins [35–38] offer an opportunity to determine if independently formed polyploids develop similar cytonuclear coordination. Previous studies have identified a myriad of genomic and transcriptomic modifications in the *Tragopogon* allopolyploids in the short time since their formation, including differential expression of homeologous loci, homeolog loss and silencing, differential proteomes [1, 39–46], and extensive chromosomal variation, such as aneuploidy and intergenomic translocations [13, 47]. Moreover, the formation of synthetic polyploids of *Tragopogon* has allowed the analysis of genomic modifications at early stages of polyploid formation [48].

Here, we use the Rubisco system (*rbcS* and *rbcL*) to examine cytonuclear coordination in naturally occurring and synthetic *Tragopogon miscellus* allopolyploids, representing independent and reciprocal formations. We characterize *rbcS* in the *Tragopogon* diploid parental species to answer the following questions: (1) How divergent are *rbcS* and *rbcL* in the *T*. *miscellus* progenitors? (2) Is there differential retention of *rbcS* homeologs in *T*. *miscellus*? (3) When both parental copies of *rbcS* are retained, do the naturally occurring and synthetic polyploids of *T*. *miscellus* show equal or biased expression of the *rbcS* homeologs?

## Materials and Methods

### Plant material

The populations sampled for *Tragopogon dubius* Scop. *T*. *pratensis* L. and *T*. *miscellus* Ownbey are listed in S1 Table, as are the synthetic lineages of *T*. *miscellus* examined. For *T*. *miscellus*, we included individuals from nine short-liguled populations (*T*. *pratensis* maternal parent) and one long-liguled population (*T*. *dubius* maternal parent), the latter representing the only extant natural population of this form. To assess potential variability in the diploid progenitors, genomic DNA and cDNA were included for multiple individuals of *T*. *dubius* (12) and *T*. *pratensis* (8) from different populations (S1 Table). For *T*. *miscellus*, four synthetic lineages (25 individuals) and ten populations (79 individuals total) were sampled. Plant material for most of the polyploids and diploids was the same as that used in Tate *et al*. [45, 46] and Buggs *et al*. [40]. For the synthetics, mature seeds were grown under standard glasshouse conditions at Massey University (Palmerston North, New Zealand); these lines were generated by Tate *et al*. [48]. For expression analyses, only a subset of individuals (31) was studied due to limited availability of fresh material for RNA extraction (S1 Table).

### DNA and RNA extraction

Both DNA and RNA were extracted from leaf tissue 28 days after seed germination. For DNA, a modified CTAB extraction protocol was used [49]. For RNA extraction, leaf tissue was flash-frozen in liquid nitrogen and ground in a 1.5-ml tube using a sterile pestle. Total RNA was extracted using the RNeasy Plant Mini kit (Qiagen, UK). First-strand cDNA was synthesized from 200 ng of total RNA using SuperScript III First-Strand Synthesis System for RT-PCR (Invitrogen, CA, USA).

### Primer design, PCR and sequencing of *rbcL* and *rbcS-1*

Full length *rbcL* was amplified from the diploid progenitors and *T*. *miscellus* using primers *rbcL1* and *rbcL2* ([50, 51] primer names as in [52]). PCR reactions were conducted in a 25-μl total volume containing 10X Thermopol buffer (New England Biolabs, USA), 10 mM dNTPs, 5 μM each primer, 0.5 Unit NEB *Taq* polymerase and ~50 ng of either genomic DNA or cDNA template. The following PCR profile was used: 95°C for 5 min, 48°C for 45 sec, 72°C for 1 min followed by 35 cycles at 95°C for 1 min, 48°C for 45 sec (2 sec added in each successive cycle) and 72°C for 1 min, with a final extension at 72°C for 10 min [52].

Initial amplification of *rbcS* was accomplished by designing PCR primers from an alignment of *T*. *dubius* ESTs (Tdu01-5MS1_K18.e, Tdu01-3MS1_B10.e, Tdu01-2Ms1_K16.e) to *Lactuca sativa rbcS* (AF162210) using Primer3 [53]. Primers (*rbcS*-2F and *rbcS*-2R) for one copy, hereafter *rbcS-1*, were used for the initial amplification of both genomic and cDNA of the diploids (Table 1). A second *rbcS*-like sequence was identified in the *T*. *dubius* EST database, but this copy is apparently a pseudo-gene as it is truncated (missing 5’ UTR through exon 1) with several premature stop codons and indels as compared to the full-length *rbcS-1* and *rbcS* sequences from other Asteraceae (S1 Fig). For this second copy, *rbcS-2*, 5’ genome walking (using methods described later for *rbcS-1*) revealed the presence of a long ~700-bp intron-like sequence (data not shown) that has not been found in any angiosperm group to date [54]. Likewise, this *rbcS-2* copy is also truncated in *T*. *pratensis*. Amplification of *rbcS-1* was conducted using the following PCR profile: 95°C for 5 min, 95°C for 1 min, 53°C for 1 min, 72°C for 1 min for 5 cycles, followed by 44 cycles of 95°C for 1 min, 48°C for 1 min, 72°C for 1 min and a final extension at 72°C for 7 min. PCR products of *rbcS-1* from genomic DNA of *T*. *dubius* and *T*. *pratensis* were cloned using the TOPO TA Cloning Kit (Invitrogen, CA, USA).

**Table 1:** *rbcS-1* primers designed in this study.

Primer Name	Experiment(s)	Primer/oligo Sequence (5’to 3’)
GS1	5’ Genome walking	ATCATACCTTCATGCACTGCACTCTTCCAC
GS2	5’ Genome walking	AGGAAAAGTCATTGGCCTTCTTGGTGACTG
AP1	5’ Genome walking	GTAATTCGCATCACTATAGCTC
AP2	5’ Genome walking	ACTATAGCTCACCGCTGGT
NA44	5’ Genome walking	GTAATTCGCATCACTATAGCTCACCGCTGGTCGACGGCCCGGGCTGGT
NA45	5’ Genome walking	PO4-ACCAGCCC-NH2
Inv. Fwd 1	3’ RACE	TGGACCTCAATCGGGTTTAT
Inv. Fwd 2	3’ RACE	CAAGAAGGAGTACCCCAACG
3'RACE adapter	3’ RACE	GACTCGAGTCGACATCG
3'RACE oligodT adapter	3’ RACE	GACTCGAGTCGACATCGATTTTTTTTTTTTTTTTTV
*rbcS-*2F	Sequencing and CAPS	AATGGCTTCCATCTCCTCCT
*rbcS-*3F	Sequencing and CAPS	TTTCCCAGTCACCAAGAAGG
*rbcS*-2R	Sequencing and CAPS	AGGCAACTTCCACATTGTCC
*rbcS*-8R	Sequencing and CAPS	CGATTGAGGTCCATCCAAAG
*rbcS*-F1	Sequencing	CAAAACATACCCATAACGTATCAGCC
*rbcS*-R3	Sequencing	AGCAGAAACATAAATTTTTATTATTATCATC
HS-Pra-Snp3	Homeolog-specific RT-PCR (forward)	AAGGCCAATGACTTTTCCTCCCGC
HS-Dub-Snp3A	Homeolog-specific RT-PCR (forward)	AAGGCCAATGACTTTTCCTCCCAT
HS-R3	Homeolog-specific RT-PCR (reverse)	CGAACATAGGCAACTTCCACATTGTCC

Ten positive clones per sample were sequenced. Prior to sequencing, PCR products were treated with exonuclease I (5 Units) and shrimp alkaline phosphatase (0.5 Unit). Cycle sequencing was performed using Big Dye v.3.1 (Applied Biosystems, Inc.), and purified products were sequenced on an ABI DNA Analyzer 3770 at the Massey Genome Service (Palmerston North, New Zealand) using both T3 and T7 plasmid primers. Sequencing results were analyzed in Sequencher v.5.1 (Gene Codes Corporation, Michigan, USA). Based on the alignment of these cloned sequences with available *T*. *dubius* ESTs, a new reverse primer (*rbcS-*8R) was designed further downstream to amplify a longer portion of *rbcS-1* from synthetic and naturally occurring *T*. *miscellus* polyploids; these longer fragments of *rbcS-1* were then sequenced directly using the aforementioned sequencing protocol with both forward (*rbcS*-2F) and reverse (*rbcS-*8R) primers (Table 1).

### Genomic and cDNA CAPS analysis

**Fig 1:**
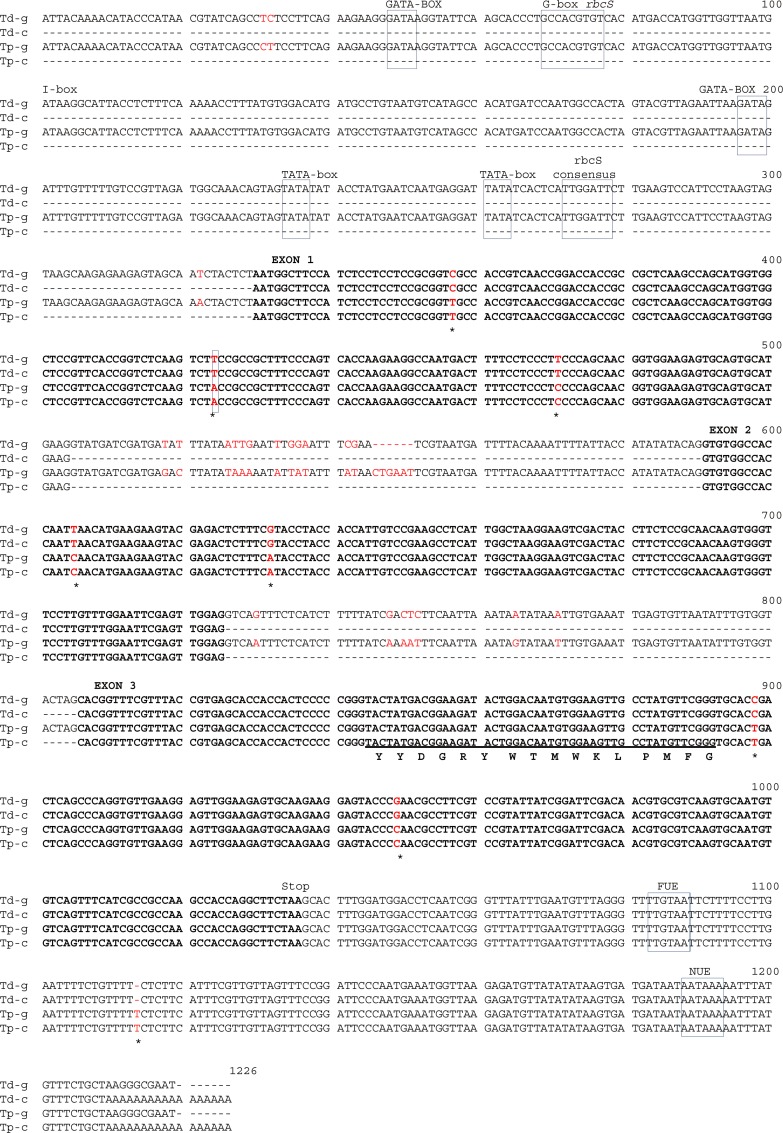
*rbcS-1 g*ene structure and locations of SNPs between *Tragopogon dubius* and *T*. *pratensis*. Upstream 5’ elements, the stop codon, and downstream 3’ elements are labelled above the nucleotide sequences. The conserved hexadecapeptide sequence [54] is highlighted and the amino acid residues indicated below the sequences. SNPs between the parental sequences are highlighted in red text and an asterisk (exons), with the one non-synonymous change surrounded by a box. Td-g = *Tragopogon dubius*-genomic; Td-c = *Tragopogon dubius*-cDNA; Tp-g = *Tragopogon pratensis*-genomic; Tp-c = *Tragopogon pratensis*-cDNA. FUE = far upstream element, NUE = near upstream element.

Sequences of *rbcS-1* for the diploid parents were aligned to determine sequence variation that could differentiate parental homeologs in *T*. *miscellus*. The programs dCAPS Finder 2.0 [55] and NEB Cutter v.1.0 [56] were used to identify diagnostic restriction sites between parental *rbcS-1* sequences. Genomic and cDNA cleaved amplified polymorphic sequence (CAPS) analyses were performed for *rbcS-1* using the forward primer *rbcS-*3F and reverse primer *rbcS-*8R (Table 1). The amplified region included exon 1 (from aligned position 433 bp), intron 1 and exon 2 (to position 1054 bp) (Fig 1). The resulting PCR products from *T*. *dubius* and *T*. *pratensis* were 462 bp for cDNA and 622 bp (*T*. *dubius*) and 628 bp (*T*. *pratensis*) from genomic DNA. PCR products were digested with *MseI*, which cuts the cDNA of *T*. *dubius* at one position (resulting in fragment sizes 375 bp and 87 bp) and does not cut *T*. *pratensis*. For genomic DNA, *T*. *dubius* is cut at three positions (resulting in fragment sizes 272 bp, 167 bp, 154 bp and 29 bp), while *T*. *pratensis* is cut at two positions (resulting in fragment sizes 327 bp, 272 bp and 29 bp). For PCRs of both genomic and cDNA, a digestion reaction was set up in a total volume of 10 μl containing 1 μl of the PCR product, 1X buffer 4 (New England Biolabs, USA), 100 μg/ml Bovine Serum Albumin and 20 Units of *MseI* enzyme (New England Biolabs, USA). Reactions were incubated at 37°C for 3 hours as specified by the manufacturer. The digested products were run on a 2% agarose gel, stained with ethidium bromide and analyzed using a Gel Doc 2000 system (Bio-Rad, UK). After establishing the protocols for the diploid parents, *rbcS-1* was PCR-amplified from the naturally occurring and synthetic polyploids of *T*. *miscellus* and digested following the same protocols. We also included an artificial hybrid DNA or cDNA template, which contained an equal mixture of the two parental DNAs or cDNAs for genomic and cDNA CAPS, respectively. As a control to verify equal expression of parental homeologs for cDNA CAPS, actin was amplified (actinF: 5’-GGAGCAGAGAGATTCCGTTG-3’, actinR: 5’-CTCTCTGGAGGAGCAACCAC-3’) and digested with *BspHI* (S2 Fig) following the CAPS protocol above. The PCR conditions used to amplify actin were the same as those for *rbcS-1*.

### 5ʹ Genome walking and 3’ RACE of *rbcS-1*

To obtain full-length *rbcS-1* sequence, we employed a 5’genome walking technique to amplify upstream unknown gene sequence (using a homemade kit following the GenomeWalker manual, Clontech Laboratories) [57]. Two outward-facing gene-specific primers were designed near the 5’ end of the *T*. *dubius rbcS-1* sequence to act as reverse primers (GS1 and GS2, Table 1). Long and short oligos to form an adapter and adapter-specific primers (to act as forward primer) were designed as described by the GenomeWalker user manual (NA44 and NA45, Table 1). *Tragopogon dubius* genomic DNA was digested with three different blunt-cutting enzymes: *EcoRV*, *ScaI* and *DraI* (New England Biolabs) independently using 2.5 μg of genomic DNA, 80 Units of restriction enzyme and 10X buffer (New England Biolabs) in a total volume of 100 μl. Reactions were incubated at 37°C for 16–18 hours. These digestion reactions were cleaned by ethanol precipitation in the presence of 20 μg glycogen and 3M sodium acetate. Adapters were ligated to the cleaned, digested genomic DNA in a total volume of 8 μl containing 25 μM adapter, 10X ligation buffer, 3 Units of T4 DNA ligase (New England Biolabs) and 0.5 μg of purified DNA. Primary PCR was conducted in 50-μl total volume using 10 mM dNTPs, 10X PCR buffer (Takara Biotechnology, Japan), 10 μM adapter primer AP1 (Forward) and gene-specific primer GS1 (Reverse) (Table 1) and 1 Unit of Takara Ex Taq polymerase (Takara Biotechnology, Japan). Cycling conditions for the primary PCR were as follows: first 7 cycles at 94°C for 25 sec, 72°C for 3 min, then the remaining 32 cycles at 94°C for 25 sec, 67°C for 3 min, then final extension at 67°C for 7 min. Primary PCR products from the first round were diluted 1:50 in ddH_2_O. In the secondary PCR, 10 μM nested or internal adapter primer AP2 (forward) and gene-specific primers GS2 (reverse) were used (Table 1), and 2 μl of diluted primary PCR product were used as template. The secondary PCR profile was as follows: 94°C for 25 sec, 72°C for 3 min for 5 cycles and 94°C for 25 sec, 67°C for 3 min for the next 20 cycles, then final extension at 67°C for 7 min. Secondary PCR products were separated on a 1% agarose gel, and products from each library were cloned and sequenced using the protocols described above. The resulting sequences were aligned to the previously obtained partial *rbcS-1* sequence of *T*. *dubius*.

To obtain the 3’ end of the *rbcS-1* gene, 3’ RACE was used. Two gene-specific nested inverse primers were designed near the 3’ end of the known *rbcS-1* gene sequence (Inv. Fwd 1 and Inv. Fwd 2, Table 1). First-strand cDNA from *T*. *dubius* was made using an oligo(dt) incorporating a 3’ RACE-specific primer sequence at the 5’ end (3’ RACE oligodT adapter, Table 1). After synthesizing *T*. *dubius* cDNA, primary PCR for 3’ RACE was conducted in a 25-μl total volume containing 5 μM gene-specific inverse primer (Inv. Fwd 2) as a forward primer and 5 μM 3’ RACE adapter primer as a reverse primer, 10X PCR buffer, 10 mM dNTPs and 1 Unit Takara Ex *Taq* polymerase. The PCR profile was as follows: 95°C for 1 min, 53°C for 1 min, 72°C for 1 min for 5 cycles, followed by 44 cycles at 95°C for 1 min, 48°C for 1 min, 72°C for 1 min, with a final extension at 72°C for 7 min. This primary PCR product was diluted 100X and used as template for nested PCR. The nested PCR mix contained all of the above reagents, except 5 μM nested primers (Inv. Fwd 1 and 3’ RACE adapter primer, Table 1) was used. Cycling conditions were the same as the 3’ RACE primary PCR. Products from the nested PCR were cloned, sequenced and aligned with the previous *rbcS-1* gene sequence from *T*. *dubius*. Once the complete *rbcS-1* gene sequence for *T*. *dubius* was obtained, new primers were designed (*rbcS*-F1 and *rbcS*-R3, Table 1) for the amplification and sequencing of the complete *rbcS-1* gene from genomic DNA and cDNA of *T*. *pratensis*.

### Prediction of *rbcS-1* gene structure

Gene structure of *rbcS-1* was predicted using Augustus (Version 2.6) [58] and GENSCAN [59]. These programs were used to confirm the transcription start site (TSS), exons, introns and other regulatory sequences as determined by cDNA sequencing of the complete *rbcS-1* gene. Plant Promoter Analysis Navigator (PlantPAN) [60] was used to identify promoter sequences of *rbcS-1*, putative transcription factor binding sites in the promoter region and conserved motifs in the promoter (S2 Table).

### Homeolog-specific RT-PCR

Homeolog-specific RT-PCR was conducted to amplify each of the diploid parental homeologs of *rbcS-1* from cDNA of the *Tragopogon miscellus* polyploids. Homeolog-specific (HS) primers were based on SNPs identified between parental *rbcS-1* homeologs [61]. Homeolog specificity was assured by adding a mismatch one nucleotide away from the 3’ end of each of the two forward HS primers (*T*. *dubius*: HS-dub-Snp3A, *T*. *pratensis*: HS-Pra-Snp3, Table 1). A common reverse primer was designed downstream of the polymorphic site (HS-R3, Table 1), corresponding to a highly conserved region in exon 3 (Fig 1). For the present experiment, forward primers were designed at the third SNP in exon 1 (corresponding to position 471 bp, Fig 1). PCR conditions were as follows: 95°C for 1 min, 60°C for 45 sec, 72°C for 1 min for 35 cycles with a final extension at 72°C for 10 min. PCR was conducted in a 25-μl total volume containing 10X PCR buffer, 10 mM dNTPs, 5 μM each primer, 0.5 Unit *Taq* polymerase (New England Biolabs, USA) and 15 ng/μl template (cDNA). The amount of template cDNA included in the PCR was quantified using a Nanodrop ND-1000 spectrophotometer (Thermo Fisher Scientific, USA) and normalized in the PCR reaction. Resulting PCR products were run on a 1.5% agarose gel and scored for presence/absence of parental homeologs or inspected to determine relative intensity of resulting bands. Different numbers of PCR cycles (30, 35, 40, and 45) were tested to determine the potential effect of amplification cycles. As no differences were observed among the different cycle numbers, the same PCR profile was used for all cDNA amplifications. Artificial hybrid cDNA, which was a 50:50 mix of *T*. *dubius* and *T*. *pratensis* cDNA, was again included as a control for equal amplification of the parental homeologs.

## Results

### *rbcS* gene family

Two *rbcS* gene copies, *rbcS-1* and *rbcS-2*, were identified from the *Tragopogon dubius* EST database. These two *rbcS* genes are fairly divergent from each other, with several SNPs, insertions and deletions in the genic regions and even more variability at the 3’ UTRs (S1 Fig). Of these two *rbcS* genes, *rbcS-1* was determined to be a functional copy, while the second *rbcS* gene, *rbcS-2*, was considered a pseudogene and a truncated copy as it had premature stop codons compared to the *rbcS-1* amino acid alignment. Several attempts at 5’genome walking experiments yielded non-*rbcS* genomic sequences (e.g., plastid *atpB* sequence) at its flanking ends where conserved sequence would have been expected. We found a similar scenario with *T*. *pratensis*. Hence, we focused on *rbcS-1* to examine potential cytonuclear coordination in *T*. *miscellus*. Sequences of *rbcS-1* sequences of *T*. *dubius* and *T*. *pratensis* were deposited in GenBank (accession numbers: KT879189, KT879190, respectively).

The total length of the *rbcS-1* sequence with coding and non-coding regions, including upstream promoter elements and downstream terminator signals, was 1212 bp in *Tragopogon dubius* and 1219 bp in *T*. *pratensis*. Fig 1 shows the full-length genomic sequences of these two diploid species with the conserved 5’ upstream elements, canonical hexadecapeptide sequence [54] and SNPs between them highlighted. No intraspecific variation in genomic sequences of *rbcS-1* was detected among individuals of either *Tragopogon dubius* or *T*. *pratensis*.

### Divergence of *rbcS-1* and *rbcL* between the diploids and their pattern of inheritance and retention in *T. miscellus*

Comparative sequence analysis of *rbcS-1* in *T*. *dubius* and *T*. *pratensis* revealed seven SNPs in the exons and a 1-bp indel in the 3’ UTR between polyadenylation signals at 1114 bp (Fig 1). Six of these SNPs were synonymous substitutions, with the second SNP at 424 bp a non-synonymous change resulting in a threonine in *T*. *pratensis* and a serine in *T*. *dubius*. Non-coding regions (upstream promoter regions and introns) were also found to contain multiple SNPs and indels (Fig 1) between the diploids. Analysis of the predicted protein structure of the *rbcS-1* sequence using the protein homology/analogy recognition engine Phyre^2^ V 2.0 [62] revealed that the non-synonymous SNP resides in an alpha-helix and does not cause any difference in predicted protein structure between *rbcS-1* parental homeologs. Genomic *rbcL* (1415 bp) sequences from both diploid parents were compared, and only one SNP was discovered at 703 bp, resulting in a synonymous substitution (Genbank accessions: KT897489, KT897491).

**Fig 2:**
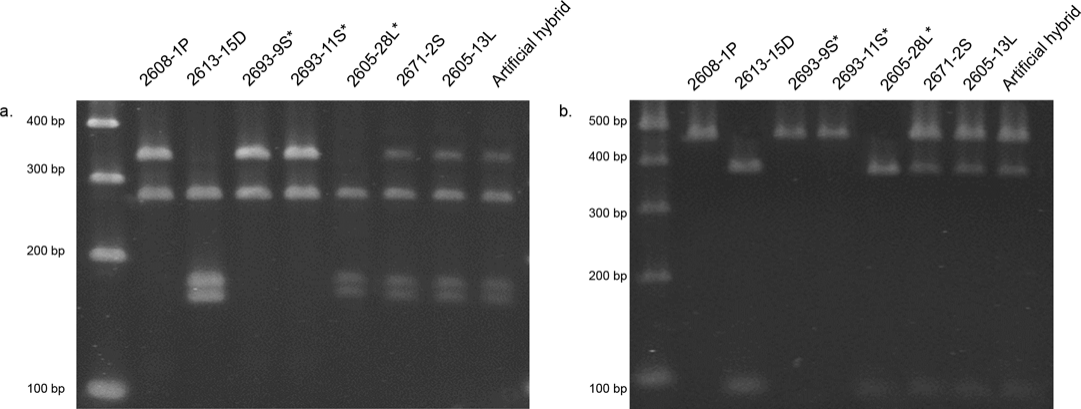
Genomic (a) and cDNA (b) cleaved amplified polymorphic sequence (CAPS) results for representative samples of naturally occurring *Tragopogon miscellus* polyploids and the diploid parents, *T*. *dubius* (D) and *T*. *pratensis* (P). *T*. *pratensis* is the maternal parent of the short-liguled (S) individuals, and *T*. *dubius* is the maternal parent of the long-liguled (L) individuals. An asterisk (*) indicates homeolog loss in *T*. *miscellus*. The artificial hybrid contained equal mixture of *T*. *dubius* and *T*. *pratensis* genomic DNA (a) or cDNA (b). Population codes are detailed in S1 Table.

To determine the pattern of retention of these subunits, genomic copies of both *rbcS-1* and *rbcL* were analyzed from 25 synthetic polyploid individuals (representing five independently generated lineages) and 79 naturally occurring polyploids from 10 populations of *T*. *miscellus*. In the case of *rbcL*, all synthetic and naturally occurring polyploids had the maternally derived sequence (i.e., *T*. *pratensis* for the short-liguled form and *T*. *dubius* for the long-liguled form; *T*. *miscellus* Genbank accessions: KT897488, KT897490). For *rbcS-1*, all synthetic polyploids and 69 of the naturally occurring polyploids had both *T*. *dubius* and *T*. *pratensis rbcS-1* homeologs, as determined by additivity of the genomic CAPS analysis (Fig 2A). Inspection of the chromatograms resulting from directly sequenced *rbcS-1* products from these same *T*. *miscellus* individuals also revealed additivity of peaks at SNPs between the parents. Ten polyploid individuals from six natural populations [Spangle (2), Garfield (1), Albion (3), Moscow (2), Pullman (1) and Troy (1)] had only one homeolog present in the genomic DNA. Of nine short-liguled individuals, six had the maternally derived *rbcS-1* homeolog (*T*. *pratensis*), and three had the paternally derived copy (*T*. *dubius*, Fig 2A, Table 2, S1 Table). One long-liguled individual from Pullman retained the *T*. *dubius* (maternal) genomic homeolog only (Fig 2A, Table 2).

**Table 2:** Naturally occurring individuals of *Tragopogon miscellus* that showed bias in the retention and/or expression of parental *rbcS-1* homeologs.

Population	Maternal parent	Lineage	Retention of *rbcS* homeologs	Expression of *rbcS* homeologs
Spangle	*T*. *pratensis*	2693–7	Both	*T*. *pratensis* > *T*. *dubius*
Spangle	*T*. *pratensis*	2693–9	*T*. *pratensis* only	*T*. *pratensis* only
Spangle	*T*. *pratensis*	2693–11	*T*. *pratensis* only	*T*. *pratensis* only
Oakesdale	*T*. *pratensis*	2671–2	Both	*T*. *pratensis* > *T*. *dubius*
Oakesdale	*T*. *pratensis*	2671–11	Both	*T*. *pratensis* > *T*. *dubius*
Garfield	*T*. *pratensis*	2688–8	*T*. *pratensis* only	*T*. *pratensis* only
Garfield	*T*. *pratensis*	2688–12	Both	*T*. *pratensis* > *T*. *dubius*
Moscow	*T*. *pratensis*	2604–17	*T*. *pratensis* only	*T*. *pratensis* only
Moscow	*T*. *pratensis*	2604–22	*T*. *pratensis* only	*T*. *pratensis* only
Moscow	*T*. *pratensis*	2604–43	Both	*T*. *pratensis* > *T*. *dubius*
Albion	*T*. *pratensis*	2625–3	*T*. *dubius* only	-
Albion	*T*. *pratensis*	2625–6	*T*. *dubius* only	-
Albion	*T*. *pratensis*	2625–8	*T*. *dubius* only	-
Troy	*T*. *pratensis*	2682–5	*T*. *pratensis* only	-
Pullman	*T*. *dubius*	2605–9	Both	*T*. *dubius* > *T*. *pratensis*
Pullman	*T*. *dubius*	2605–28	*T*. *dubius* only	*T*. *dubius* only
Pullman	*T*. *dubius*	2605–46	Both	*T*. *dubius* > *T*. *pratensis*

A dash (-) indicates that material was not available to study a particular individual for both retention (genomic DNA) and expression (cDNA).

### Expression of *rbcS-1* homeologs in *T*. *miscellus* polyploids

**Fig 3:**
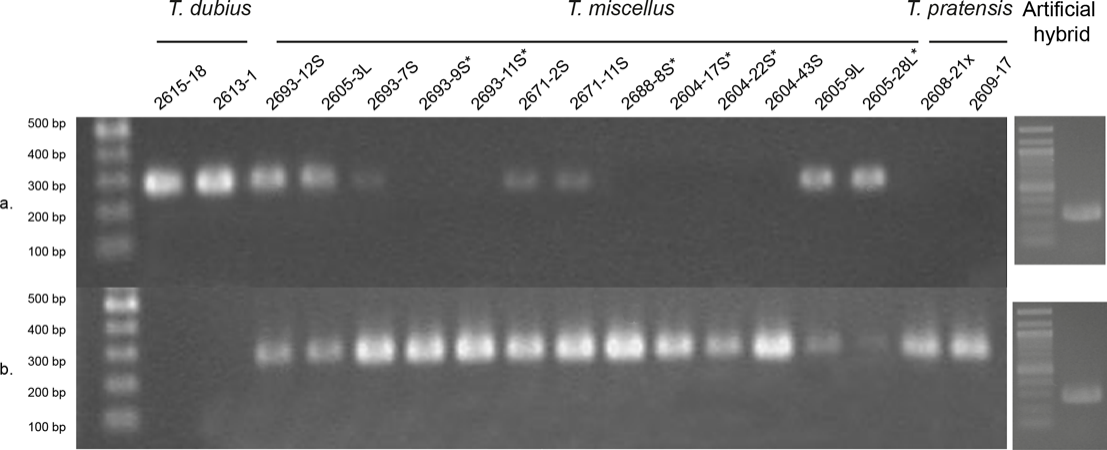
Homeolog-specific HS-RT-PCR of *rbcS-1* for representative individuals of *Tragopogon miscellus* and diploid progenitors *T*. *dubius* and *T*. *pratensis*. RT-PCR results using *T*. *dubius*-specific primers (a) and *T*. *pratensis*-specific primers (b). *T*. *pratensis* is the maternal parent of the short-liguled (S) individuals, and *T*. *dubius* is the maternal parent of the long-liguled (L) individuals. An asterisk (*) indicates homeolog loss in *T*. *miscellus*. The artificial hybrid contained equal mixture of *T*. *dubius* and *T*. *pratensis* cDNA. Population codes are detailed in S1 Table.

Relative expression of parental *rbcS-1* homeologs was determined by cDNA CAPS (Fig 2B) and HS-RT-PCR (Fig 3) analyses. Because the cDNA CAPS results did not appear to show any appreciable differences in homeolog expression, HS-RT-PCR was employed as a potentially more sensitive method to detect differential homeolog expression. For the six synthetic polyploid individuals examined, all showed equal expression of the parental homeologs (S1 Table). Of the 31 naturally occurring polyploid individuals examined, five showed deviation from additive expression (Table 2, Figs 2B and 3), as determined by comparison of the relative intensity or presence/absence of parental homeologs in the polyploids, using the positive controls as a baseline for those comparisons. In all cases where both genomic copies were detected, the maternally derived *rbcS-1* homeolog was expressed at a greater level (Fig 3). The five individuals showing differential expression were from different populations and represented one long-liguled individual from Pullman and four short-liguled individuals [Moscow (1), Oakesdale (2), and Spangle (1)]. All *T*. *miscellus* individuals that showed biased expression of the *rbcS-1* homeologs were found to have equal expression of *T*. *dubius* and *T*. *pratensis* actin homeologs (S2 Fig).

## Discussion

### Characterization of *rbcS-1* in *Tragopogon* diploid species

In angiosperms, the rbcS small subunit is fairly divergent among species and is often encoded by a multigene nuclear family [63–67], compared to the plastid *rbcL*, which is highly conserved and present in single copy [66]. Indeed, only one SNP distinguished the *Tragopogon* progenitor *rbcL* copies, and this resulted in a synonymous substitution. As is the case in most other eudicots [30, 63], the *rbcS-1* gene in *Tragopogon* consists of three exons separated by two short introns. The second copy found in *T*. *dubius* and *T*. *pratensis* (*rbcS-2*) may represent a pseudogene on its way to being lost from the genome. In most other angiosperms, the *rbcS* gene family ranges in size from four (*Arabidopsis*) to more than 22 (wheat) copies [64, 67, 68]. Generally, only one or two members of the *rbcS* gene family are strongly expressed in the angiosperms surveyed to date, and these genes contribute more than half of the total *rbcS* transcripts [69, 70]. Indeed, concerted evolution of the *rbcS* gene family is probably a common phenomenon even in diploid taxa [30]. In other members of Asteraceae, *Lactuca sativa* (tribe Cichorieae) has six *rbcS* genes [71], and *Flaveria* species (Heliantheae) contain from 5–16 genes [72], while *Helianthus* (Heliantheae) [73] and *Chrysanthemum* (Anthemideae) [69] each has only one *rbcS* gene. Thus, *rbcS* may be diverse in copy number even within the same plant family or tribe (*Tragopogon* is a member of the Cichorieae), perhaps due to general processes of gene loss, genome downsizing, concerted evolution or a mere lack of expansion of the gene family [74, 75].

Interspecific *rbcS-1* sequence variation was low between the parental diploids *Tragopogon dubius* and *T*. *pratensis* (2.5% sequence divergence, 0.5% amino acid divergence), compared to other genera (*Gossypium* [31], *Arabidopsis* [64], *Triticum* [68]). Only one non-synonymous substitution was detected between *T*. *dubius* and *T*. *pratensis rbcS-1* homeologs; this SNP resided in the α-helix of the predicted protein and did not result in a change in protein structure or folding. It is not obvious if this change has an influence on the seemingly maternal bias toward homeolog retention and expression in the *T*. *miscellus* polyploids.

### Genomic loss and expression of *rbcS-1* homeologs biased towards the maternal parent in *T. miscellus* polyploids

From an evolutionary perspective, the dynamic nature of polyploid genomes is well known [8, 76, 77]. Homeolog loss is one genetic modification commonly observed following genome duplication in a diverse array of polyploid species (*Brassica* [11], *Triticum* [14], *Tragopogon* [44], *Gossypium* [78], *Arabidopsis* [79]). The results presented here are generally consistent with previous findings of preferential retention and expression of maternal homeologs in *Tragopogon* [40, 44, 45, 80]. In polyploids, homeolog losses may be associated with dosage compensation to efficiently maintain gene regulatory mechanisms [81]. In the case of cytonuclear coordination involving multi-subunit complexes, like Rubisco, loss of the paternal homeolog and retention of the maternal copy may facilitate the regulatory coordination between the maternal and paternal genomes. However, in *Tragopogon*, this coordination is not immediate upon polyploid formation as the synthetics and most of the naturally occurring polyploids still retain and express both parental homeologs. Compared to other polyploid systems that are much older [30, 31], we also did not find any evidence for unique mutations (autapomorphies) in the natural or synthetic *Tragopogon miscellus rbcS* sequences. Examination of other cytonuclear complexes would lend insight to the potential to maintain genomic balance between nuclear homeologs and their cytoplasmic counterparts.

Changes in duplicate gene expression are another consequence of allopolyploidization [1, 8, 82, 83], which may involve biased expression of the parental homeologs in the polyploids. This bias may be balanced, with an equal number of genes showing bias towards each parent, or unbalanced, with more genes displaying bias towards one parent [28, 84–87]. Previous studies on *Tragopogon* identified alterations in expression of homeologous loci (i.e., *T*. *dubius* loci silenced more often than *T*. *pratensis*) [1, 44, 88]. In this study, expression of parental *rbcS-1* homeologs was biased toward the maternal parent, although again very few individuals showed this pattern and it was not immediately upon allopolyploid formation.

The successful establishment of F_1_ hybrids and allopolyploids requires coordination between the maternally inherited cytoplasmic (plastid and mitochondrial) and the biparentally inherited nuclear genomes to facilitate genomic stability [22, 89]. Cytoplasmic factors, including a variety of nucleo-cytoplasmic co-evolutionary pathways, have been considered responsible for post-zygotic hybrid incompatibilities and therefore a driver of plant speciation [23]. Here we show that this coordination may be a slower process and does not occur immediately upon formation in *Tragopogon*, however, given that the naturally occurring *Tragopogon miscellus* populations are less than 80 years old (~40 generations as they are biennials), sorting out potential cytonuclear incompatibilities may only take a few generations to begin. Examination of the synthetic lineages over successive generations would lend valuable insight as to when these changes start to occur.

The biased retention and expression of maternal *rbcS-1* homeologs in individuals from different populations indicates repeatability of this evolutionary trajectory because each population of *T*. *miscellus* represents an independent formation [33, 36, 38, 90]. However, within a population, the observed homeolog losses may result from the same historical event; thus, our estimates of absolute losses may be lower (six rather than ten). Although the majority of the individuals showed maternal bias, three individuals from the Albion population were an exception. These short-liguled individuals retained the paternal (*T*. *dubius*) *rbcS-1* genomic homeolog, instead of the *T*. *pratensis* copy. Unfortunately, fresh material was not available to study *rbcS-1* expression in individuals from this population, so we do not know if the paternal bias is restricted to genome loss or extends to homeolog expression as well for individuals that retained both homeologs. In a previous study of homeolog loss in *T*. *miscellus* [45], this population showed a greater number of homeolog losses (individual 2625–3 in particular) than all other populations. Although in general there seems to be a recurrent pattern toward maternal bias, in some populations different *rbcS/rbcL* parental combinations might be beneficial to facilitate cytonuclear interactions.

Given that the predicted protein structure of both parental *rbcS-1* homeologs is the same and the *rbcL* progenitor copies only differ by one synonymous SNP, exactly what has driven differential expression of the maternal copy of *rbcS-1* in the naturally occurring polyploids is not yet understood. There are several possible explanations for the expression biases observed. First, the polymorphisms observed between *rbcS-1* homeologs in the promoter region (e.g., one SNP was found eight nucleotides away from the transcription start site) might be responsible for differential regulation of *rbcS-1* homeologs, and later, their interaction with the *rbcL*-encoded subunit. Dean *et al*. [63] found that specific *rbcS* copies in *Petunia* (Solanaceae) contained ‘enhancer-like’ elements in the promoter region that resulted in quantitative differences in expression levels, even when there was a high degree of similarity in coding sequence among other copies. This region, termed box II, was also identified in other solanaceous genera (tomato, *Solanum*, and tobacco, *Nicotiana*), and *rbcS* copies with this motif were expressed at a greater level than were other copies. *Tragopogon* also contains this enhancer-like motif, but no SNPs between the parents were identified in this region. Perhaps the other polymorphisms in the promoter region contribute to the expression differences observed here.

A second explanation for the expression bias is that the one non-synonymous change in exon I (threonine in *T*. *pratensis* and serine in *T*. *dubius*) may result in differential selection on the *rbcS-1* copies under some conditions. Further research involving protein-protein interactions between rbcS/rbcL subunits in *T*. *miscellus* would be helpful to clarify the complexities of these cytonuclear interactions.

## Supporting Information

S1 Fig Alignment of *rbcS* cDNA sequences and protein translations for *Tragopogon dubius*, *T*. *pratensis* and selected Asteraceae. The conserved hexadecapeptide motif (YYDGRYWTMWKLPMFG) is indicated in red text.
(PDF)

S2 Fig cDNA-CAPS of actin to verify equal expression of parental copies in *T*. *miscellus* polyploids. A.H. stands for artificial hybrid which was a 1:1 mixture of *T*. *dubius* and *T*. *pratensis* cDNA.
(PDF)

S1 Table Data summary for all *Tragopogon miscellus* allotetraploids and diploid parents (*T*. *dubius* and *T*. *pratensis*) examined. Data are summarized from genomic DNA and cDNA CAPS and homeolog-specific RT-PCR. Note: Letters “D” and “P” correspond to the diploid parents *T*. *dubius* and *T*. *pratensis*, respectively. A ‘D’ or a ‘P’ indicates that only one parental homeolog was detected in genomic DNA or expressed. P>D indicates that the *T*. *pratensis* homeolog showed higher relative expression than the *T*. *dubius rbcS-1* homeolog in the *T*. *miscellus* individual and vice versa for D>P.
(PDF)

S2 Table Transcription factor binding sites identified in the *Tragopogon rbcS-1* promoter region. (PDF)
