# Supplementary material for: Cytonuclear Coordination Is Not Immediate upon Allopolyploid Formation in Tragopogon miscellus (Asteraceae) Allopolyploids
Source: PLoS One. 2015 Dec 8;10(12):e0144339. doi: 10.1371/journal.pone.0144339 (PMC4673006; doi:10.1371/journal.pone.0144339)
Supplement: S1 Fig — The conserved hexadecapeptide motif (YYDGRYWTMWKLPMFG) is indicated in red text. (PDF) [file pone.0144339.s001.pdf]

S1 Fig. Alignment of *rbcS-1* and *rbcS-2* cDNA sequences from *Tragopogon dubius*, *T. pratensis* and other Asteraceae representatives along with protein translation for both genes. The conserved hexadecapeptide motif (YYDGRYWTMWKLPMFG) is indicated in red text.

```
                                                          60
Tdubius_rbcS1_cDNA              AATGGCTTCCATCTCCTCCTCCGCGGTCGCCACCGTCAACCGGACCACCGCCGCTCAAGC
                                 M  A  S  I  S  S  S  A  V  A  T  V  N  R  T  T  A  A  Q  A
Tdubius_rbcS2_cDNA              ------------------------------------------------------------

Tpratensis_rbcS2-cDNA           ------------------------------------------------------------

Lactuca_sativaD14001            AATGGCCTCCATCTCCTCCTCAGCCATCGCCACCGTCAACCGGACCACCTCCACCCAAGC
                                 M  A  S  I  S  S  S  A  I  A  T  V  N  R  T  T  S  T  Q  A
Flaveria_pringleiU29933         AATGGCTTCGATCCCCGCTACCGT---CGCCACCGTCGC---------------TCAAGC
                                 M  A  S  I  P  A  T  V     A  T  V  A                 Q  A
Flaveria_pringleiU29934         AATGGCTTCGATCTCCTCCACCGT---CGCCACCGTCAGCCGGGCCGCCCCCGCTCAAGC
                                 M  A  S  I  S  S  T  V     A  T  V  S  R  A  A  P  A  Q  A
Flaveria_pringleiU29936         AATGGCTTCGATCTCCTCCACCGT---CGCCACCGTCAGCCGGGCCGCCCCCGCTCAAGC
                                 M  A  S  I  S  S  T  V     A  T  V  S  R  A  A  P  A  Q  A
Helianthus_annuusX05079         AATGGCTTCGATCTCCTCCTCAGT---CGCGACCGTTAGCCGGACCGCCCCTGCTCAGGC
                                 M  A  S  I  S  S  S  V     A  T  V  S  R  T  A  P  A  Q  A

                                                          120
Tdubius_rbcS1_cDNA              CAGCATGGTGGCTCCGTTCACCGGTCTCAAGTCTTCCGCCGCTTTCCCAGTCACCAAGAA
                                 S  M  V  A  P  F  T  G  L  K  S  S  A  A  F  P  V  T  K  K
Tdubius_rbcS2_cDNA              ------------------------------------------------------------

Tpratensis_rbcS2-cDNA           ------------------------------------------------------------

Lactuca_sativaD14001            TAGCTTGGCAGCTCCATTCACCGGCCTCAAGTCTAACGTAGCTTTCCCAGTTACCAAGAA
                                 S  L  A  A  P  F  T  G  L  K  S  N  V  A  F  P  V  T  K  K
Flaveria_pringleiU29933         CAACATGGTGGCTCCGTTCACCGGTCTTAAGGCCAATGCTGCCTTCCCCGTCACCAAGAA
                                 N  M  V  A  P  F  T  G  L  K  A  N  A  A  F  P  V  T  K  K
Flaveria_pringleiU29934         CAACATGGTCGCCCCGTTCACCGGTCTTAAGTCCAACGTCGCCTTCCCCGCCACCAAGAA
                                 N  M  V  A  P  F  T  G  L  K  S  N  V  A  F  P  A  T  K  K
Flaveria_pringleiU29936         CAACATGGTCGCCCCGTTCACCGGTCTTAAGTCCAACGCCGCCTTCCCCGCCACCAAAAA
                                 N  M  V  A  P  F  T  G  L  K  S  N  A  A  F  P  A  T  K  K
Helianthus_annuusX05079         CAACATGGTGGCTCCGTTCACCGGCCTTAAGTCCAACGCCGCCTTCCCCACCACCAAGAA
                                 N  M  V  A  P  F  T  G  L  K  S  N  A  A  F  P  T  T  K  K

                                                          180
Tdubius_rbcS1_cDNA              GGC---CAATGACTTTTCCTCCCTTCCCAGCAACGGTGGAAGAGTGCAGTGCATGAAGGT
                                 A        N  D  F  S  S  L  P  S  N  G  G  R  V  Q  C  M  K  V
Tdubius_rbcS2_cDNA              ------------------------------------------------------------

Tpratensis_rbcS2-cDNA           ------------------------------------------------------------

Lactuca_sativaD14001            GGCTAACAATGACTTTTCATCCCTACCCAGCAACGGTGGAAGAGTACAATGCATGAAGGT
                                 A  N  N  D  F  S  S  L  P  S  N  G  G  R  V  Q  C  M  K  V
Flaveria_pringleiU29933         GGT---CAACGACTTCTCCACCCTTCCCAGCAACGGTGGAAGAGTGCAATGCATGAAGGT
                                 V        N  D  F  S  T  L  P  S  N  G  G  R  V  Q  C  M  K  V
Flaveria_pringleiU29934         GGC---TAACGACTTCTCCACCCTTCCTAGCAACGGTGGAAGAGTGCAATGCATGAAGGT
                                 A        N  D  F  S  T  L  P  S  N  G  G  R  V  Q  C  M  K  V
Flaveria_pringleiU29936         GGC---TAACGACTTCTCCACCCTTCCCAGCAACGGTGGAAGAGTGCAATGCATGAAGGT
                                 A        N  D  F  S  T  L  P  S  N  G  G  R  V  Q  C  M  K  V
Helianthus_annuusX05079         GGC---TAACGACTTCTCCACCCTTCCCAGCAACGGTGGAAGAGTTCAATGCATGAAGGT
                                 A        N  D  F  S  T  L  P  S  N  G  G  R  V  Q  C  M  K  V

                                                          240
Tdubius_rbcS1_cDNA              GTGGCCACCAATTAACATGAAGAAGTACGAGACTCTTTCGTACCTACCACCATTGTCCGA
                                 W  P  P  I  N  M  K  K  Y  E  T  L  S  Y  L  P  P  L  S  E
Tdubius_rbcS2_cDNA              ------------------------------------------------------------

Tpratensis_rbcS2-cDNA           ------------------------------------------------------------

Lactuca_sativaD14001            GTGGCCACCAATTGGGTTGAAGAAGTACGAGACTCTTTCATACCTACCACCATTGTCCGA
```

```
                            W  P  P  I  G  L  K  K  Y  E  T  L  S  Y  L  P  P  L  S  D
Flaveria_pringleiU29933    GTGGCCACCACTTGGAAAGAAGAAGTACGAGACTCTTTCATACCTTCCAGACCTAACTGA
                            W  P  P  L  G  K  K  K  Y  E  T  L  S  Y  L  P  D  L  T  E
Flaveria_pringleiU29934    GTGGCCACCACTTGGAAAGAAGAGGTACGAGACTCTTTCATACCTTCCAAATCTAACTGA
                            W  P  P  L  G  K  K  R  Y  E  T  L  S  Y  L  P  N  L  T  E
Flaveria_pringleiU29936    GTGGCCACCACTTGGAAAGAAGAAGTACGAGACTCTTTCATACCTTCCAAATCTAACTGA
                            W  P  P  L  G  K  K  K  Y  E  T  L  S  Y  L  P  N  L  T  E
Helianthus annuusX05079    GTGGCCACCACTTGGATTGAAGAAGTACGAGACTCTCTCATACTTACCACCACTAACTGA
                            W  P  P  L  G  L  K  K  Y  E  T  L  S  Y  L  P  P  L  T  E

                                                                                       300
Tdubius_rbcS1_cDNA         AGCCTCATTGGCTAAGGAAGTCGACTACCTTCTCCGCAACAAGTGGGTTCCTTGTTTGGA
                            A  S  L  A  K  E  V  D  Y  L  L  R  N  K  W  V  P  C  L  E
Tdubius_rbcS2_cDNA         --------GGTCTAAGGAAGTTGACTACCTTCTCCGC-ACAAATGGGTTCCTTGCCTAGA
                                     V  *  G  S  *  L  P  S  P  H  K  W  V  P  C  L  E
Tpratensis_rbcS2_cDNA      ---------------------------------CCGC-ACAAATGGGTTCCTTGCCTAGA
                                                             P  H  K  W  V  P  C  L  E
Lactuca_sativaD14001       CGAGGCATTGTCTAAGGAAATCGACTACCTTATCCGCAACAAGTGGATTCCATGCTTGGA
                            E  A  L  S  K  E  I  D  Y  L  I  R  N  K  W  I  P  C  L  E
Flaveria_pringleiU29933    GGTCCAGTTGGCTAAGGAAGTCGACTACCTGCTCCGCAACAAATGGGTTCCTTGTTTGGA
                            V  Q  L  A  K  E  V  D  Y  L  L  R  N  K  W  V  P  C  L  E
Flaveria_pringleiU29934    GGCCCAGTTGGCTAAGGAAGTCGACTACCTCCTCCGCAACAAATGGGTTCCTTGTTTGGA
                            A  Q  L  A  K  E  V  D  Y  L  L  R  N  K  W  V  P  C  L  E
Flaveria_pringleiU29936    GGCCCAGTTGGCTAAGGAAGTCGACTACCTCCTCCGCAACAAATGGGTTCCTTGTTTGGA
                            A  Q  L  A  K  E  V  D  Y  L  L  R  N  K  W  V  P  C  L  E
Helianthus annuusX05079    AACTCAGTTGGCTAAGGAAGTCGACTACTTGCTCCGCAAAAAATGGGTTCCTTGTTTGGA
                            T  Q  L  A  K  E  V  D  Y  L  L  R  K  K  W  V  P  C  L  E

                                                                                       360
Tdubius_rbcS1_cDNA         ATTCGAGTTGGAGCACGGTTTCGTTTACCGTGAGCACCACCACTCCCCCGGGTACTATGA
                            F  E  L  E  H  G  F  V  Y  R  E  H  H  H  S  P  G  Y  Y  D
Tdubius_rbcS2_cDNA         ATTCGAGTTGGAGCATGGTTTCGTCTACCGTGAGCACCACCACTCCCCCGGATACTATGA
                            F  E  L  E  H  G  F  V  Y  R  E  H  H  H  S  P  G  Y  Y  D
Tpratensis_rbcS2_cDNA      ATTCGAGTTGGAGCATGGTTTCGTCTACCGTGAGCACCACCACTCCCCCGGATACTATGA
                            F  E  L  E  H  G  F  V  Y  R  E  H  H  H  S  P  G  Y  Y  D
Lactuca_sativaD14001       ATTCGAGTTGGAGCATGGTTTCGTTTACCGTGAGCACCACCATTCCCCCGGATACTATGA
                            F  E  L  E  H  G  F  V  Y  R  E  H  H  H  S  P  G  Y  Y  D
Flaveria_pringleiU29933    ATTCGAGTTGGAGCACGGTTTTGTCTACCGTGAGAACGCAAGATCTCCCGGATACTATGA
                            F  E  L  E  H  G  F  V  Y  R  E  N  A  R  S  P  G  Y  Y  D
Flaveria_pringleiU29934    ATTCGAGTTGGAGCACGGTTTTGTCTATCGCGAGAACGCAAGATCTCCCGGATACTATGA
                            F  E  L  E  H  G  F  V  Y  R  E  N  A  R  S  P  G  Y  Y  D
Flaveria_pringleiU29936    ATTCGAGTTGGAGCACGGTTTTGTCTATCGCGAGAACGCAAGATCTCCCGGATACTATGA
                            F  E  L  E  H  G  F  V  Y  R  E  N  A  R  S  P  G  Y  Y  D
Helianthus annuusX05079    ATTCGAGTTGGAGCACGGTTTTGTCTACCGTGAGAACGCCAGATCCCCCGGATACTATGA
                            F  E  L  E  H  G  F  V  Y  R  E  N  A  R  S  P  G  Y  Y  D

                                                                                       420
Tdubius_rbcS1_cDNA         CGGAAGATACTGGACAATGTGGAAGTTGCCTATGTTCGGGTGCACCGACTCAGCCCAGGT
                            G  R  Y  W  T  M  W  K  L  P  M  F  G  C  T  D  S  A  Q  V
Tdubius_rbcS2_cDNA         TGGCCGATACTGGACAATGTGGAAGTTGCCTATGTTCGGGTGCACTGATTCAGCCCAGGT
                            G  R  Y  W  T  M  W  K  L  P  M  F  G  C  T  D  S  A  Q  V
Tpratensis_rbcS2_cDNA      TGGCCGATACTGGACAATGTGGAAGTTGCCTATGTTCGGGTGCACTGACTCAGCCCAGGT
                            G  R  Y  W  T  M  W  K  L  P  M  F  G  C  T  D  S  A  Q  V
Lactuca_sativaD14001       TGGAAGATACTGGACAATGTGGAAGTTGCCTATGTTCGGATGCACTGACTCAGCTCAGGT
                            G  R  Y  W  T  M  W  K  L  P  M  F  G  C  T  D  S  A  Q  V
Flaveria_pringleiU29933    CGGAAGATACTGGACAATGTGGAAATTGCCTATGTTCGGATGCACCGACTCAGCCCAAGT
                            G  R  Y  W  T  M  W  K  L  P  M  F  G  C  T  D  S  A  Q  V
Flaveria_pringleiU29934    CGGAAGATACTGGACAATGTGGAAGTTGCCTATGTTCGGATGCACCGACTCAGCCCAAGT
                            G  R  Y  W  T  M  W  K  L  P  M  F  G  C  T  D  S  A  Q  V
Flaveria_pringleiU29936    CGGAAGATACTGGACAATGTGGAAGTTGCCTATGTTCGGATGCACCGACTCAGCCCAAGT
                            G  R  Y  W  T  M  W  K  L  P  M  F  G  C  T  D  S  A  Q  V
Helianthus annuusX05079    CGGAAGATACTGGACAATGTGGAAATTGCCTATGTTCGGTTGCACCGACTCAGCCCAAGT
                            G  R  Y  W  T  M  W  K  L  P  M  F  G  C  T  D  S  A  Q  V

                                                                                       480
Tdubius_rbcS1_cDNA         GTTGAAGGAGTTGGAAGAGTGCAAGAAGGAGTACCCGAACGCCTTCGTCCGTATTATCGG
                            L  K  E  L  E  E  C  K  K  E  Y  P  N  A  F  V  R  I  I  G
Tdubius_rbcS2_cDNA         GTTGAAGGAGCTTGAAGAGTGCAAGAAGGAGTACCCCAACGCCTTCGTCCGTATCATCGG
                            L  K  E  L  E  E  C  K  K  E  Y  P  N  A  F  V  R  I  I  G
Tpratensis_rbcS2_cDNA      GTTGAAGGAGCTTGAAGAGTGCAAGAAGGAGTACCCCAACGCCTTCGTCCGTATCATCGG
                            L  K  E  L  E  E  C  K  K  E  Y  P  N  A  F  V  R  I  I  G
```

```
Lactuca_sativaD14001          CATGAAGGAGGTTGGAGAGTGCAAGAAGGAGTACCCCAACGCCTTCATCCGTGTTATTGG
                               M  K  E  V  G  E  C  K  K  E  Y  P  N  A  F  I  R  V  I  G
Flaveria_pringleiU29933       GATGAAGGAGCTTCAAGAGTGCAAGAAGGAGTACCCTCAGGCATGGATCCGTATCATTGG
                               M  K  E  L  Q  E  C  K  K  E  Y  P  Q  A  W  I  R  I  I  G
Flaveria_pringleiU29934       GATGAAGGAGCTTCAAGAATGCAAGAAGGAGTACCCTCAGGCATGGATCCGTATCATCGG
                               M  K  E  L  Q  E  C  K  K  E  Y  P  Q  A  W  I  R  I  I  G
Flaveria_pringleiU29936       GATGAAGGAGCTTCAAGAATGCAAGAAGGAGTACCCTCAGGCATGGATCCGTATCATCGG
                               M  K  E  L  Q  E  C  K  K  E  Y  P  Q  A  W  I  R  I  I  G
Helianthus annuusX05079       GATGAAGGAGCTTGCTGAATGCAAGAAGGAGTACCCCCAGGCCTGGATCCGTATCATCGG
                               M  K  E  L  A  E  C  K  K  E  Y  P  Q  A  W  I  R  I  I  G


                                                                                         540
Tdubius_rbcS1_cDNA            ATTCGACAACGTGCGTCAAGTGCAATGTGTCAGTTTCATCGCCGCCAAGCCACCAGGC--
                               F  D  N  V  R  Q  V  Q  C  V  S  F  I  A  A  K  P  P  G
Tdubius_rbcS2_cDNA            ATTCGACAACGTTCGCCAAGTGCAATGTGTCAGCTTCATCGCTGCCAAACCACCAGGC--
                               F  D  N  V  R  Q  V  Q  C  V  S  F  I  A  A  K  P  P  G
Tpratensis_rbcS2_cDNA         ATTCGACAACGTTCGCCAAGTGCAATGTGTCAGCTTCATCGCTGCCAAACCACCAGGC--
                               F  D  N  V  R  Q  V  Q  C  V  S  F  I  A  A  K  P  P  G
Lactuca_sativaD14001          ATTCGACAACATTCGTCAAGTGCAATGTATCAGTTTCATCGTTGCCAAGCCCCCAGGCGT
                               F  D  N  I  R  Q  V  Q  C  I  S  F  I  V  A  K  P  P  G  V
Flaveria_pringleiU29933       ATTTGACAACGTTCGTCAAGTGCAATGCATCAGTTTCATTGCCTCAAAACCAGGTGGC--
                               F  D  N  V  R  Q  V  Q  C  I  S  F  I  A  S  K  P  G  G
Flaveria_pringleiU29934       ATTTGACAACGTTCGTCAAGTTCAGTGCATCAGTTTCATCGCTTCCAAGCCAGAAGGC--
                               F  D  N  V  R  Q  V  Q  C  I  S  F  I  A  S  K  P  E  G
Flaveria_pringleiU29936       ATTTGACAACGTTCGTCAAGTTCAATGTATCAGTTTCATTGCCTCCAAACCAGATGGC--
                               F  D  N  V  R  Q  V  Q  C  I  S  F  I  A  S  K  P  D  G
Helianthus annuusX05079       ATTTGACAATGTTCGTCAAGTTCAATGTATCATGTTCATTGCTTCCAGGCCAGATGGT--
                               F  D  N  V  R  Q  V  Q  C  I  M  F  I  A  S  R  P  D  G

                                    547
Tdubius_rbcS1_cDNA            -TTCTAA
                               F  *
Tdubius_rbcS2_cDNA            -TTCTAA
                               F  *
Tpratensis_rbcS2_cDNA         -TTCTAA
                               F  *
Lactuca_sativaD14001          TCTCTAA
                               L  *
Flaveria_pringleiU29933       -TTCTAA
                               F  *
Flaveria_pringleiU29934       -TTCTAA
                               F  *
Flaveria_pringleiU29936       -TTCTAA
                               F  *
Helianthus annuusX05079       -TACTAA
                               Y  *
```
