# Supplementary material for: Cytonuclear Coordination Is Not Immediate upon Allopolyploid Formation in Tragopogon miscellus (Asteraceae) Allopolyploids
Source: PLoS One. 2015 Dec 8;10(12):e0144339. doi: 10.1371/journal.pone.0144339 (PMC4673006; doi:10.1371/journal.pone.0144339)
Supplement: S2 Fig — A.H. stands for artificial hybrid which was a 1:1 mixture of T. dubius and T. pratensis cDNA. (PDF) [file pone.0144339.s002.pdf]

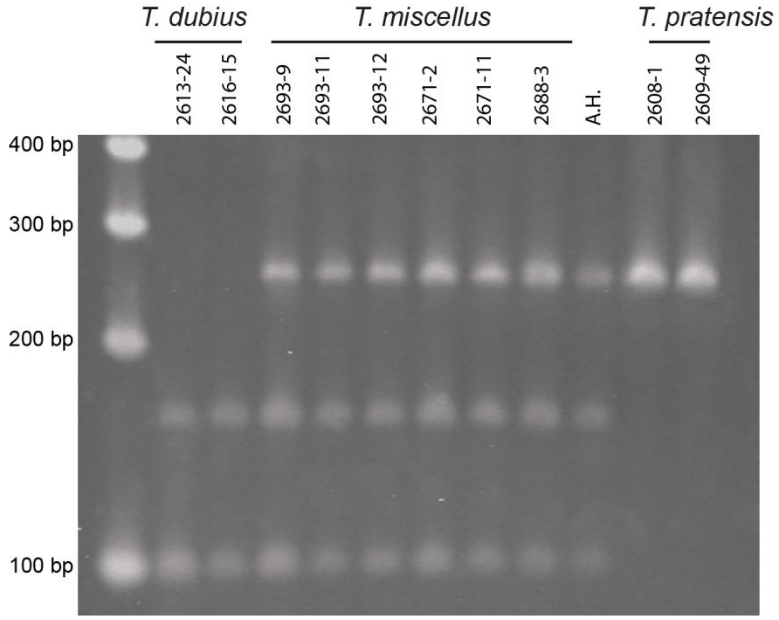

S2 Fig. Representative cDNA-CAPS of actin as a control to verify equal expression of parental homeologs in the *T. miscellus* allopolyploids that showed biased maternal expression for *rbcS-1*. A.H. stands for artificial hybrid which was a 1:1 mixture of *T. dubius* and *T. pratensis* cDNA.
