# Supplementary material for: Cytonuclear Coordination Is Not Immediate upon Allopolyploid Formation in Tragopogon miscellus (Asteraceae) Allopolyploids
Source: PLoS One. 2015 Dec 8;10(12):e0144339. doi: 10.1371/journal.pone.0144339 (PMC4673006; doi:10.1371/journal.pone.0144339)
Supplement: S1 Table — Data are summarized from genomic DNA and cDNA CAPS and homeolog-specific RT-PCR. Note: Letters “D” and “P” correspond to the diploid parents T. dubius and T. pratensis, respectively. A ‘D’ or a ‘P’ indicates that only one parental homeolog was detected in genomic DNA or expressed. P>D indicates that the T. pratensis homeolog showed higher relative expression than the T. dubius rbcS-1 homeolog in the T. miscellus individual and vice versa for D>P. (PDF) [file pone.0144339.s003.pdf]

S1 Table. List of naturally occurring and synthetic polyploids (*T. miscellus*) and diploid parents (*T. dubius* and *T. pratensis*) examined.

Data are summarized from genomic DNA and cDNA CAPS and homeolog-specific RT-PCR. Note: Letters "D" and "P" correspond to the diploid parents *T. dubius* and *T. pratensis*, respectively. A 'D' or a 'P' indicates that only one parental homeolog was detected in genomic DNA or expressed. P>D indicates that the *T. pratensis* homeolog showed higher relative expression than the *T. dubius rbcS-1* homeolog in the *T. miscellus* individual and vice versa for D>P.

Species	Population /synthetic generation	Maternal parent (for polyploid)	Unique no./ Synthetic line	Lineage	Retention	Expression
Diploid species						
T. dubius	Spokane			2615-22	D	D
T. dubius	Spokane			2615-4	D	D
T. dubius	Spangle		1987	2616-8	D	D
T. dubius	Spangle		1988	2616-11	D	D
T. dubius	Oakesdale		1980	2670-9	D	D
T. dubius	Oakesdale		1981	2670-10	D	D
T. dubius	Garfield		1938	2687-3	D	D
T. dubius	Garfield		1943	2687-11	D	D
T. dubius	Pullman		1073	2613-1	D	D
T. dubius	Pullman		1095	2613-35	D	D
T. dubius	Troy			2683-1	D	D
T. dubius	Troy			2683-7	D	D
T. pratensis	Spangle		2138	2692-1	P	P
T. pratensis	Spangle		2139	2692-2	P	P
T. pratensis	Oakesdale		2150	2672-4	P	P
T. pratensis	Oakesdale		2151	2672-5	P	P
T. pratensis	Garfield		2136	2689-15	P	P
T. pratensis	Garfield		2137	2689-17	P	P

T. pratensis	Moscow		1049	2608-31	P	P
T. pratensis	Moscow			2608-35X	P	P
Short-liguled form						
T. miscellus	Spokane	*T. pratensis*		2664-3	Both	-
T. miscellus	Spokane	*T. pratensis*		2664-5a	Both	-
T. miscellus	Spokane	*T. pratensis*		2664-5b	Both	-
T. miscellus	Spokane	*T. pratensis*		2664-6	Both	-
T. miscellus	Spokane	*T. pratensis*		2617-1	Both	-
T. miscellus	Spokane	*T. pratensis*		2617-4	Both	-
T. miscellus	Spokane	*T. pratensis*		2617-6	Both	-
T. miscellus	Spokane	*T. pratensis*		2617-7	Both	-
T. miscellus	Spokane	*T. pratensis*		2617-8	Both	-
T. miscellus	Spokane	*T. pratensis*		2617-9	Both	-
T. miscellus	Spokane	*T. pratensis*		2617-12	Both	-
T. miscellus	Spokane	*T. pratensis*		2617-21	Both	-
T. miscellus	Spangle	*T. pratensis*	2121	2693-3	Both	-
T. miscellus	Spangle	*T. pratensis*	2123	2693-5	Both	-
T. miscellus	Spangle	*T. pratensis*	2125	2693-7	Both	P>D
T. miscellus	Spangle	*T. pratensis*	2126	2693-8	Both	Both
T. miscellus	Spangle	*T. pratensis*	2127	2693-9	P	P
T. miscellus	Spangle	*T. pratensis*	2129	2693-11	P	P
T. miscellus	Spangle	*T. pratensis*	2130	2693-12	Both	Both
T. miscellus	Spangle	*T. pratensis*	2131	2693-13	Both	-
T. miscellus	Spangle	*T. pratensis*	2132	2693-14	Both	-
T. miscellus	Spangle	*T. pratensis*	2133	2693-15	Both	-
T. miscellus	Rosalia	*T. pratensis*		2667-3	Both	-
T. miscellus	Rosalia	*T. pratensis*		2667-4	Both	-
T. miscellus	Oakesdale	*T. pratensis*	2055	2671-1	Both	-
T. miscellus	Oakesdale	*T. pratensis*	2056	2671-2	Both	P>D
T. miscellus	Oakesdale	*T. pratensis*	2057	2671-3	Both	-
T. miscellus	Oakesdale	*T. pratensis*	2058	2671-4	Both	Both
T. miscellus	Oakesdale	*T. pratensis*	2061	2671-7	Both	Both
T. miscellus	Oakesdale	*T. pratensis*	2063	2671-9	Both	-

T. miscellus	Oakesdale	*T. pratensis*	2064	2671-10	Both	-
T. miscellus	Oakesdale	*T. pratensis*	2065	2671-11	Both	P>D
T. miscellus	Garfield	*T. pratensis*	2099	2688-1	Both	-
T. miscellus	Garfield	*T. pratensis*	2100	2688-2	Both	Both
T. miscellus	Garfield	*T. pratensis*	2103	2688-5	Both	-
T. miscellus	Garfield	*T. pratensis*	2104	2688-6	Both	Both
T. miscellus	Garfield	*T. pratensis*	2106	2688-8	P	P
T. miscellus	Garfield	*T. pratensis*		2688-10	Both	Both
T. miscellus	Garfield	*T. pratensis*	2109	2688-11	Both	Both
T. miscellus	Garfield	*T. pratensis*	2110	2688-12	Both	Both
T. miscellus	Garfield	*T. pratensis*	2111	2688-13	Both	-
T. miscellus	Albion	*T. pratensis*		2625-1	Both	-
T. miscellus	Albion	*T. pratensis*		2625-2	Both	-
T. miscellus	Albion	*T. pratensis*		2625-3	D	-
T. miscellus	Albion	*T. pratensis*		2625-5	Both	-
T. miscellus	Albion	*T. pratensis*		2625-6	D	-
T. miscellus	Albion	*T. pratensis*		2625-8	D	-
T. miscellus	Albion	*T. pratensis*		2625-9	Both	-
T. miscellus	Albion	*T. pratensis*		2625-10	Both	-
T. miscellus	Moscow	*T. pratensis*	1154	2604-4	Both	-
T. miscellus	Moscow	*T. pratensis*	1157	2604-10	Both	-
T. miscellus	Moscow	*T. pratensis*	1158	2604-11	Both	Both
T. miscellus	Moscow	*T. pratensis*	1162	2604-15	Both	Both
T. miscellus	Moscow	*T. pratensis*		2604-17	P	P
T. miscellus	Moscow	*T. pratensis*	1171	2604-20	Both	Both
T. miscellus	Moscow	*T. pratensis*	1173	2604-22	P	P
T. miscellus	Moscow	*T. pratensis*	1176	2604-24	Both	-
T. miscellus	Moscow	*T. pratensis*	1186	2604-35	Both	-
T. miscellus	Moscow	*T. pratensis*	1402	2604-43	Both	P>D
T. miscellus	Troy	*T. pratensis*		2682-1	Both	-
T. miscellus	Troy	*T. pratensis*		2682-2	Both	-
T. miscellus	Troy	*T. pratensis*		2682-3	Both	-
T. miscellus	Troy	*T. pratensis*		2682-4	Both	-

T. miscellus	Troy	*T. pratensis*		2682-5	P	-
T. miscellus	Troy	*T. pratensis*		2682-6	Both	-
T. miscellus	Troy	*T. pratensis*		2682-7	Both	-
T. miscellus	Troy	*T. pratensis*		2682-11	Both	-
T. miscellus	Troy	*T. pratensis*		2682-12	Both	-
Long-liguled form						
T. miscellus	Pullman	*T. dubius*		2605-3	Both	Both
T. miscellus	Pullman	*T. dubius*	1194	2605-4	Both	-
T. miscellus	Pullman	*T. dubius*	1196	2605-7	Both	Both
T. miscellus	Pullman	*T. dubius*		2605-9	Both	D>P
T. miscellus	Pullman	*T. dubius*	1203	2605-13	Both	Both
T. miscellus	Pullman	*T. dubius*	1204	2605-14	Both	Both
T. miscellus	Pullman	*T. dubius*	1210	2605-24	Both	Both
T. miscellus	Pullman	*T. dubius*	1211	2605-28	D	D
T. miscellus	Pullman	*T. dubius*	1212	2605-29	Both	Both
T. miscellus	Pullman	*T. dubius*	1216	2605-42	Both	Both
T. miscellus	Pullman	*T. dubius*	1221	2605-46	Both	D>P
Synthetic polyploids						
T. miscellus	S_0	*T. pratensis*	111-1		Both	-
T. miscellus	S_1	*T. pratensis*	111-1-7		Both	-
T. miscellus	S_1	*T. pratensis*	111-1-8		Both	-
T. miscellus	S_1	*T. pratensis*	111-1-23		Both	Both
T. miscellus	S_0	*T. pratensis*	111-4		Both	-
T. miscellus	S_1	*T. pratensis*	111-4-4		Both	-
T. miscellus	S_1	*T. pratensis*	111-4-17		Both	Both
T. miscellus	S_0	*T. pratensis*	111-5		Both	-
T. miscellus	S_1	*T. pratensis*	111-5-7		Both	-
T. miscellus	S_1	*T. pratensis*	111-5-9		Both	-
T. miscellus	S_1	*T. pratensis*	111-5-18		Both	Both
T. miscellus	S_0	*T. pratensis*	111-7		Both	-
T. miscellus	S_1	*T. pratensis*	111-7-11		Both	Both
T. miscellus	S_1	*T. pratensis*	67-2-1		Both	-

T. miscellus	S_1	*T. pratensis*	67-2-4		Both	-
T. miscellus	S_1	*T. pratensis*	67-2-7		Both	Both
T. miscellus	S_1	*T. pratensis*	67-3-6		Both	-
T. miscellus	S_1	*T. pratensis*	67-3-8		Both	-
T. miscellus	S_0	*T. pratensis*	79-1		Both	-
T. miscellus	S_1	*T. pratensis*	79-1-3		Both	-
T. miscellus	S_1	*T. pratensis*	79-11-3		Both	-
T. miscellus	S_0	*T. dubius*	119-2		Both	-
T. miscellus	S_0	*T. dubius*	129-7		Both	-
T. miscellus	S_1	*T. dubius*	129-7-1		Both	-
T. miscellus	S_1	*T. dubius*	129-7-14		Both	Both
