# Supplementary material for: Cytonuclear Coordination Is Not Immediate upon Allopolyploid Formation in Tragopogon miscellus (Asteraceae) Allopolyploids
Source: PLoS One. 2015 Dec 8;10(12):e0144339. doi: 10.1371/journal.pone.0144339 (PMC4673006; doi:10.1371/journal.pone.0144339)
Supplement: S2 Table — (PDF) [file pone.0144339.s004.pdf]

S1 Table. Transcription factor binding sites in *Tragopogon rbcS-1* promoter region as determined by Plant Promoter Analysis Navigator (PlantPAN).

Transcription factor	Site	Sequence	Species
AGL3	11	atacCCATAacgtatcag	*Arabidopsis*
Athb-1	254	tgaggATTATatca	*Arabidopsis*
ATHB-9	91	ttggttaATGATaaggcat	*Arabidopsis*
CDC5	22	gtaTCAGCctc	*Arabidopsis*
PIF3	65	ccctgcCACGTgtcacat	*Arabidopsis*
ABRELATED1	72	ACGTG	*Arabidopsis*
ANAERO1CONSENSUS	202	TTTGTTT	Maize/*Arabidopsis*/Pea/Barley/Rice
CACGTGMOTIF	71	CACGTG	Tomato/*Arabidopsis*/Snapdragon/Wheat
Core	1	ATTA	*Arabidopsis*
Core	108	ATTA	*Arabidopsis*
GATABOX	100	GATA	*Petunia/Arabidopsis/*Rice
GATABOX	196	GATA	*Petunia/Arabidopsis/*Rice
GATABOX	263	TATC	*Petunia/Arabidopsis/*Rice
GBOXLERBCS	69	GCCACGTGT	Tomato*/Arabidopsis*
GT1CONSENSUS	108	ATTACC	Pea/Oat/Rice/Tobacco/*Arabidopsis*
HY5AT	68	TGCCACGTGTCA	*Arabidopsis*
IBOXCORENT	100	GATAAGG	Tobacco
IBOXCORENT	48	GATAAGG	tobacco
IBOX	100	GATAAG	Tomato/*Arabidopsis*
IBOX	48	GATAAG	Tomato*/Arabidopsis*
LRENPCABE	68	TGCCACGT	Tobacco
MYB2COREATCYCB1	213	CCGTT	*Arabidopsis*
RBCSCONSENSUS	272	TTGGATT	Tomato*/petunia*/Tobacco/Pea

SORLIP1AT	158	GCCAC	*Arabidopsis*
SORLIP1AT	69	GCCAC	*Arabidopsis*
WBOXNTERF3	82	TGACC	Tobacco
